# Post–13-Valent Pneumococcal Conjugate Vaccine Dynamics in Young Children 

**DOI:** 10.3201/eid2708.210037

**Published:** 2021-08

**Authors:** Corinne Levy, Naim Ouldali, Emmanuelle Varon, Stéphane Béchet, Stéphane Bonacorsi, Robert Cohen

**Affiliations:** Association Clinique et Thérapeutique Infantile du Val-de-Marne, Créteil, France (C. Levy, N. Ouldali, S. Béchet, R. Cohen);; Groupe de Pathologie Infectieuse Pédiatrique, Paris, France (C. Levy, N. Ouldali, E. Varon, S. Bonacorsi, R. Cohen);; Université Paris Est, IMRB-GRC GEMINI, Créteil (C. Levy, E. Varon, R. Cohen);; Centre Hospitalier Intercommunal de Créteil, Créteil (C. Levy, E. Varon, R. Cohen);; Association Française de Pédiatrie Ambulatoire, Saint-Germain-en-Laye, France (C. Levy, R. Cohen);; Assistance Publique-Hôpitaux de Paris, Hôpital Robert Debré, ECEVE INSERM UMR 1123, Paris (N. Ouldali, S. Bonacorsi);; Université de Paris, IAME, UMR 1137, INSERM, Paris (S. Bonacorsi)

**Keywords:** pneumoccocal serotypes, children, PCVs, pneumococcal conjugate vaccines, *Streptococcus pneumoniae*, vaccines, France

**To the Editor:** We read with interest the article by Ben-Shimol et al. (*1*), which described the disproportionate increase of non–13-valent pneumococcal conjugate vaccine (PCV) additional PCV20 serotypes (vaccine type [VT] 20–13) in patients who had respiratory infections or invasive pneumococcal disease (IPD) after PCV13 implementation in Israel. The authors emphasized the higher disease potential of VT20–13 serotypes compared with non-VT20 serotypes. We would like to complement their results with data from France and highlight the similarities and the differences in serotype distribution. Our long-term prospective population-based surveillance comprises pneumococcal isolates from 793 healthy carriers, 4,474 acute otitis media patients, and 441 IPD patients, all of whom were children <24 months of age (*2*–*4*). We found that VT13 serotypes accounted for 8%, VT20–13 for 30%, and non-VT20 for 60% of infections in healthy carriers and acute otitis media patients during 2015–2018. Like Ben-Shimol et al. (*1*), we found that the most common VT20–13 serotypes were 15B/C and 11A, and the most common non-VT20 serotypes were 23B, 15A, and 35B.

From the early PCV13 (2009–2011 in both countries) to late PCV13 period (2015–2017 in Israel and 2015–2018 in France), the prevalence of IPD caused by VT13 serotypes declined by ≈90% in both countries. However, VT20–13 serotypes predominated in Israel, whereas non-VT20 serotypes predominated in France. Although Israel had higher proportions of serotypes 12F (26.9% vs. 0.9%) and 33F (10.6% vs. 4.4%) than France (*1*), France saw the emergence of the non-VT20 serotype 24F (27.4%) during 2015–2018. This emergent serotype led to a higher proportion of PCV20 serotypes in Israel (62%) than in France (41.6%). The differences in vaccine type distribution between the 2 countries were mainly based on the very high rates of serotypes 12F and 33F in Israel and the emerging serotype 24F in France (*5*). Apart from these serotypes, the serotype distribution in IPD was very similar (Table).

**Table:** Comparison of pneumococcal serotypes in children <24 mo of age in Israel, 2015–2017, and France, 2015–2018*

Serotypes	Cases of invasive pneumococcal disease, no. (%)	
	Israel	France
Total	216 (100.0)	113 (100.0)
PCV13 serotypes	22 (10.2)	5 (4.4)
22F	8 (3.7)	8 (7.1)
33F	23 (10.6)	5 (4.4)
PCV15 serotypes†	53 (24.5)	18 (15.9)
8	2 (0.9)	2 (1.8)
10A	8 (3.7)	11 (9.7)
11A	3 (1.4)	2 (1.8)
12F	58 (26.9)	1 (0.9)
15B/C	10 (4.6)	13 (11.5)
PCV20 serotypes‡	134 (62.0)	47 (41.6)
Non-VT20	82 (38.0)	66 (58.4)
24F	7 (3.2)	31 (27.4)
VT20–13	112 (51.9)	42 (37.2)
Non-PCV13	194 (89.8)	108 (95.6)

*Non-VT20, serotypes in PCV20; PCV13, 13-valent PCV; PCV15, 15-valent PCV; PCV20, 20-valent PCV; PCV, pneumococcal conjugate vaccine; VT20–13, vaccine types in PCV20 but not PCV13. †Comprises PCV13 serotypes as well as 22F and 33F. ‡Comprises PCV15 serotypes as well as 8, 10A, 11A, 12F, and 15B/C.

In conclusion, data from Israel and France show a similar effect of PCV13 on the distribution of VT13 serotypes. The role of emerging non-PCV13 serotypes in carriage was also similar. However, we observed unexpected discrepancies in the serotype replacement pattern, driven by few highly invasive non-PCV13 serotypes. This finding suggests that serotype replacement during the PCV13 era is complex and multifactorial, and has implications for the expected effects of next-generation PCVs. Finally, France and Israel had similar serotype distributions that differed in only the late PCV13 period as a result of the emergence of some invasive specific clones (*5*–*8*).
